# Idiopathic Renal Infarction: An Important Differential Diagnosis of Unexplained Flank Pain

**DOI:** 10.7759/cureus.18206

**Published:** 2021-09-23

**Authors:** Ali S Al-Shareef, Emad Alwafi, Mohammed Alzailaie, Bader Shirah

**Affiliations:** 1 Emergency Medicine, King Abdulaziz Medical City, Jeddah, SAU; 2 Research Office, King Abdullah International Medical Research Center, Jeddah, SAU; 3 College of Medicine, King Saud bin Abdulaziz University for Health Sciences, Jeddah, SAU; 4 Internal Medicine, King Abdulaziz Medical City, Jeddah, SAU; 5 Critical Care Medicine, King Abdulaziz Medical City, Jeddah, SAU

**Keywords:** thrombosis, renal artery, renal, infarction, flank pain, acute, abdomen

## Abstract

Unexplained flank pain should alert physicians regarding the possibility of acute renal infarction. Despite its rare occurrence, prompt diagnosis and management of renal infarction can improve outcomes. We report a previously healthy 37-year-old male who presented to the emergency department complaining of left flank pain. Computed tomography angiogram showed a thrombus in the left renal artery. The patient responded well to treatment with anticoagulation, and the symptoms resolved. The present case conforms with other experiences of good outcomes when treatment is initiated in a timely manner. Anticoagulation led to resolution of the thrombus and restoration of perfusion. This case report should remind physicians to consider renal infarction in the differential diagnosis of an acute abdomen patient with no risk factors.

## Introduction

Idiopathic renal infarction is an extremely rare condition [1]. Of patients seen at the emergency department (ED), only 0.007% are diagnosed with acute renal infarction. Cases are usually due to thromboembolism, which usually originates from the heart, aorta, or in situ thrombosis due to underlying hypercoagulable condition or injury to or dissection of a renal artery [2]. Unfortunately, renal infarction is usually missed in the ED as patients present with nonspecific abdominal pain that mimics a wide differential of nonspecific abdominal pain, which is the most common cause of admission in the ED [3]. Moreover, it has been shown that the time to diagnosis of renal infarction following presentation is often more than two days with <50% of patients being diagnosed promptly [4]. In this report, we present a case of flank pain presenting to the ED and discuss the evaluation and overall treatment options.

## Case presentation

A 37-year-old gentleman, with no known chronic medical illness, presented to the ED with a sudden onset of severe left flank pain that radiates to his testicles of 7-hour duration. He described the pain as colicky, severe, and exacerbated by movement. Of note, he also reported decreased urinary frequency and volume. He denied any other symptoms, including fever, nausea, vomiting, trauma, dysuria, and hematuria. He is a smoker but denies any use of alcohol or illicit drugs.

The initial ED evaluation included an electrocardiogram that showed normal sinus rhythm and rate. His physical examination showed normal vital signs, including body temperature of 37.0 °C, blood pressure of 138/80 mmHg, heart rate of 77 beats per minute, respiratory rate of 20 breaths per minute, and pulse oximetry reading of 99% on room air. He had a body mass index of 20.8 kg/m^2^. He appeared in moderate distress due to pain. Abdominal examination showed tenderness to palpation in the left flank and left upper quadrant. Other system examinations were normal.

His laboratory assessment showed a trace of hematuria in urine dipstick without gross hematuria, leukocytosis 12.1 × 10^9^/L, hemoglobin 13.0 g/dL, blood urea nitrogen 4.4 mmol/L, creatinine of 83 umol/L, and lipase 19 U/L. Lactic acid was normal at 1.02, and coagulation profile was normal with international normalized ratio 1.1, partial thromboplastin time 34 seconds, and prothrombin time 13 seconds.

**Figure 1:**
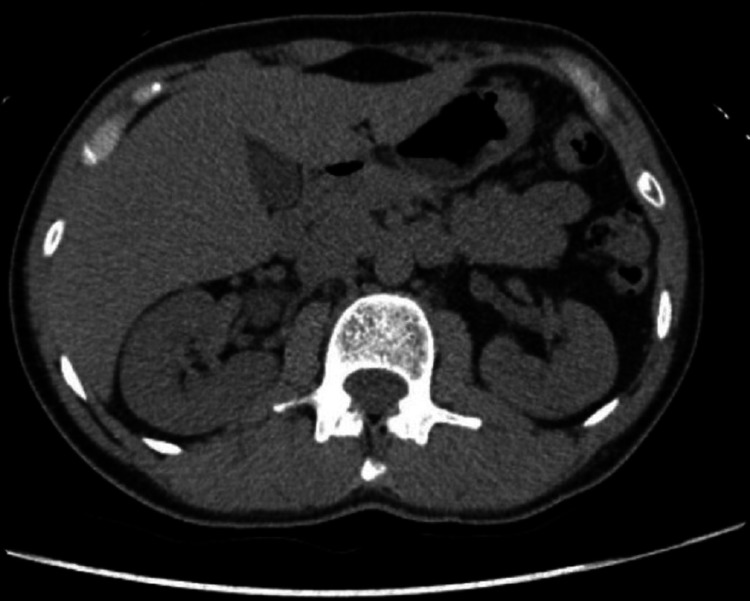
CT scan of the abdomen showing that both kidneys are in normal size and position, and the urinary tract is not obstructed. CT, computed tomography

Nephrolithiasis was strongly considered as a cause of the patient's pain based on the nature of the pain as well as the presence of hematuria. Therefore, non-enhanced helical computed tomography (CT) scan of the abdomen was obtained. The CT showed both kidneys normal in size and position, and the urinary tract was not obstructed (Figure 1).

The patient's pain improved with analgesia. As his workup, including non-enhanced CT, was reassuring, he was discharged and given instructions when to come back to ED as well as given an appointment with a primary care physician for follow-up.

**Figure 2:**
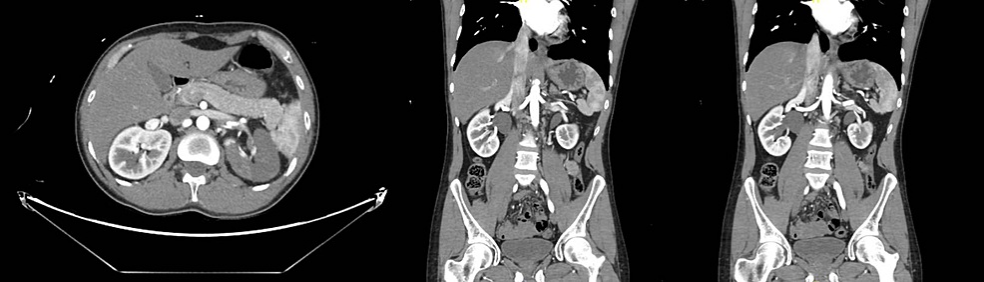
Contrast-enhanced CT scan of the abdomen showing a left renal infarction. CT, computed tomography

The patient presented back to the ED 10 hours later with the same pain but in a continuous unrelenting fashion. A contrast-enhanced CT scan of the abdomen revealed a left renal infarction due to left renal artery thromboembolic disease (Figure 2).

Following this finding, the appropriate services were consulted and recommended treatment with anticoagulation. He was started on normal saline and morphine for pain control as well as an unfractionated heparin bolus of 80 mg/kg and an infusion at 18 mg/kg/h. Furthermore, the patient was admitted for workup of thrombophilic conditions.

His echocardiogram was normal and further laboratory workup was negative, including antinuclear antibodies and rheumatoid factor. No definite underlying thrombophilic condition was found. Apart from smoking nicotine, no other causes nor risk factors were found. His pain resolved on the third day and he was discharged on warfarin as anticoagulation with hematology follow-up for therapeutic monitoring. 

## Discussion

There are several causes of renal infarction including cardioembolic, renal artery injury, hypercoagulable state, and idiopathic. In a published retrospective study, 27/94 patients with renal infarction were identified as idiopathic [5]. Early diagnosis of renal infarction is challenging to physicians due to its nonspecific clinical presentation, which is shared with other common diseases. Nausea, vomiting, fever, and flank pain are the main symptoms that occur with nephrolithiasis and pyelonephritis. The most common symptoms among patients with renal infarction were abdominal pain (53%), flank pain (50%), acute elevation of blood pressure at initial presentation (48%), nausea (16.9%), vomiting (13%), and fever (10%) [5].

Lactate dehydrogenase (LDH) may be used as a marker for renal infarction since LDH occurs only in renal infarction rather than nephrolithiasis and pyelonephritis. A high LDH concentration was frequently associated with renal infarction in 90.5% of patients [5-8]. Other causes of elevated serum LDH such as hemolysis and myocardial infarction can be easily excluded if there is no significant increase in aspartate transaminase and alkaline phosphatase levels as well as specific markers of cardiac injury such as troponin I [9]. Other laboratory evaluations include creatinine elevation as a marker of kidney injury, which occurs in 40.4% of patients [5]. Urinalysis on admission revealed hematuria in 21/39 (54%) and proteinuria in 17/38 (45%) [6].

The next step is performing a CT scan of the abdomen without contrast to exclude nephrolithiasis. Then, a contrast-enhanced CT scan to evaluate for infarction. After diagnosis, the challenge of the selection of modality of treatment is lower in comparison to the diagnosis dilemma. Management includes screening for thrombophilia, antiplatelets, anticoagulants, and percutaneous endovascular therapy.

## Conclusions

Unexplained flank pain should alert physicians regarding the possibility of acute renal infarction. Despite its rare occurrence, prompt diagnosis and management of renal infarction can improve outcomes. This case report should remind physicians to consider renal infarction in the differential diagnosis of an acute abdomen patient with no risk factors. The present case conforms with other experiences of good outcomes when treatment is initiated in a timely manner.
